# A review on silver-mediated DNA base pairs: methodology and application

**DOI:** 10.1186/s40824-022-00254-w

**Published:** 2022-03-07

**Authors:** Qiao Sun, Xiao Xie, Yujie Song, Litao Sun

**Affiliations:** 1 SEU-FEI Nano-Pico Center, Key Laboratory of MEMS of Ministry of Education, School of Electronic Engineering, Southeast University, Nanjing, 210096 China; 2 Center for Advanced Materials and Manufacture, Southeast University-Monash University Joint Research Institute, Suzhou, 215123 China

**Keywords:** DNA, Metal-mediated base pairs, Bioinorganic chemistry, Coordination chemistry

## Abstract

The investigation of the interaction between metal ions and DNA has always attracted much attention in the fields of bioinorganic chemistry, supramolecular coordination chemistry, and DNA nanotechnology. Its mode of action can be simply divided into two aspects. On the one hand, it is non-specific electrostatic adsorption, mainly including Na^+^, K^+^, Mg^2+^, Ca^2+^ and other physiologically regulating ions; on the other hand, it is specific covalent binding, such as Pt^2+^, Hg^2+^, Ag^+^ and other heavy metal ions. This article focuses on the mechanism of action between Ag^+^ and DNA mismatch pair C-C, and summarizes its main characterization methods and various applications. It aims to provide a certain reference for the field of biological devices. With the development of cryo-electron microscopy and liquidcell TEM, the structure of C-Ag^+^-C is expected to be further characterized, which will be more widely used.

## Introduction

In recent decades, diseases caused by bacterial infections have seriously threatened human health and become a global public health problem. Therefore, comprehensive interest has been put on antibacterial materials [1]. Antibacterial materials are generally divided into two categories: organic and inorganic material [2]. Organic antibacterial materials mainly include phenols, alcohols, halogenated compounds, quaternary ammonium salts, and nature-derived materials such as chitosan. Their bactericidal effects are remarkable, but many of them are highly toxic, poorly heat-resistant, and easy to decompose. Their antibacterial mechanism is relatively simple—combining with the anions on the cell membrane surface of bacteria and molds, or reacting with sulfhydryl groups to suppress the synthesis of protein and cell membranes, and inhibit the reproduction of bacteria and molds. With the unreasonable use of these antibacterial materials, many bacteria have rapidly evolved and developed corresponding drug resistance [1]. Typical inorganic antibacterial materials include metal ions (Ag^+^, Cu^2+^, Zn^2+^), metal oxides, and metals combined with phosphates. They usually have better heat stability, a prolonged active time, and they are not easy to cause resistance because of the complex antibacterial mechanism. Thereby inorganic materials are given more and more attention in all aspects.

Silver as well as its compounds has been used for antibacterial and treatment since thousands of years, which is the most widely studied metal among the others [3]. Ancient Greeks and Romans fabricated silverware to store water, food and wine to avoid spoilage. Silver was used to treat ulcers and promote wound healing by Hippocrates [4], and silver nitrate was also applied to wound care and surgical disinfection. In the epoch of nanotechnology, silver nanomaterials have broad antibacterial spectrum and high activity [5]. Even at extremely low concentrations, they can effectively kill various pathogens, therefore there have been a large range of applications in medical, civil and household supplies.

There have been numerous studies on the antibacterial properties of silver, but its specific mechanism has not been fully understood [5]. Mainstream understandings include [6–8]: a) the adhesion of Ag to the cell wall and cell membrane surface can cause membrane damage and changes in molecular transport; b) it can penetrate into the cell and destroy intracellular structures (mitochondria, vacuoles, ribosomes) and biological molecules (proteins, lipids, DNA) thus affect cell functions; c) it can induce the production of reactive oxygen species (ROS) and free radicals which are toxic to cells; d) it can regulate cell signaling systems, leading to cell death.

Although a proper amount of silver has a significant antibacterial effect, excessive silver exposure is toxic to many organisms [9]. It has been reported that silver with a concentration higher than 1.6 nM is toxic to fish and microorganisms. Inhalation through breathing, intravenous injection can also cause harm to the human body [10]. The affected organs and systems may include eyes, kidneys, skin, nerves, respiratory system, immune system, hepatobiliary system and reproductive system. For example, silver precipitation caused by long-term exposure to silver and its compounds can induce blue-gray skin [11]. Therefore, the US Environmental Protection Agency stipulates that the maximum pollution of silver in drinking water cannot exceed 100 μg/L [12, 13].

Nowadays, it has been found that the toxicity of heavy metals in the environment is mainly caused by the covalent binding of heavy metal ions and nucleic acids. Besides, the interaction between heavy metals and nucleic acids is also the basis for the development of anticancer drugs, for example the well-known anti-cancer drug cisplatin [14] (cis-[Pt(NH_3_)_2_Cl_2_]). Studies have shown that Pt^2+^ can bind to guanine bases on the DNA chain to promote cancer cell death. Therefore, the design of anticancer drugs similar to cisplatin calls for in-depth understandings of the interaction process between Pt^2+^ (and other metal ions) and nucleic acids.

Up to now, most of the proposed research results are based on experimental investigations, which fell on the spatial dimension of cell or micrometer. The nanoscale mechanism, e. g. molecular interactions, is yet to be more deeply investigated.

Although the research on the combination of Ag^+^ and DNA is still primitive, many applications based on the principle of specific binding have been developed, such as metal ion detectors, logic gates and switches, DNA mutation detection and nanowires. This review first introduces the specific binding mode of silver ions and DNA, as well as the typical researches. Then we describe the applications of the combination of silver ion and DNA in device and biological regimes in the past ten years. Finally, the future of the development of the interaction between silver and DNA is proposed.

## Metal-mediated base pairs

### Non-specific combination of DNA and metal ions

The basic unit of DNA is a deoxynucleotide, which is composed of one molecule of phosphoric acid, one deoxyribose and one nitrogenous base. The B-DNA double helix model proposed by Watson and Crick in 1953 [15] laid the foundation for the development of modern molecular biology, which leads biology into the molecular scale. In the double helix structure, two anti-parallel polynucleotide strands are coiled into a helical structure driven by hydrogen bonds between complementary A-T and C-G base pairs, and aromatic bases. The helix is stabilized by the π-π stacking effect between π electrons and the electrostatic force between the negative charge on the phosphate group and the cation in the dispersive medium. Under physiological conditions, most of the phosphoric acid on DNA releases protons, making DNA negatively charged. The negatively charged phosphate skeleton can easily stabilize its structure by non-specific binding with metal ions such as Na^+^, K^+^ and Mg^2+^ or Ca^2+^ in the human body.

### Specific combination of Pt^2+^ and DNA

The binding between metal ions and DNA bases has also aroused extensive attention [16–19]. Pt^2+^ in the known anticancer drug cisplatin [14] can bind to the N7 position of the two bases of DNA guanine and adenine to completely de-stack the purine ring and enhance the stability between base pairs. These effects cause DNA to bent significantly in the cisplatin binding site and cause the DNA double helix to unwind, thereby effectively inhibiting DNA transcription and ultimately leading to cell death.

### T-Hg^2+^-T

However, in 2006, it was discovered that the metal ion-Hg^2+^ can combine with double-stranded DNA to form metal-base pair structure without affecting the double-strand conformation. Mercury with high toxicity can cause the denaturation of the DNA double-stranded structure. However, in 2006, Miyake [20] reported that double-stranded DNA containing T-T base mismatches became more stable under the action of Hg^2+^. By 1D 1H NMR it is found that the double-strand DNA containing T-T mispairing has a resonance peak in the imino proton region in the absence of Hg^2+^. The resonance in the imino proton region weakens with the concentration of Hg^2+^ and finally almost disappears when the molecular ratio is 1:1, which proves that Hg^2+^ is directly connected to the N3 site of thymine. In addition, the van der Waals force radius of mercury is ~ 1.44 Å, and the base pair spacing in the double-stranded DNA is ~ 3.4 Å, which indicates that the insertion of mercury will not destruct the double helix. The hydrogen bond between base pairs in the double-stranded DNA is replaced by a metal base bond to form a metal-base pair complex.

### Research on the structure of C-Ag^+^-C

**Fig. 1:**
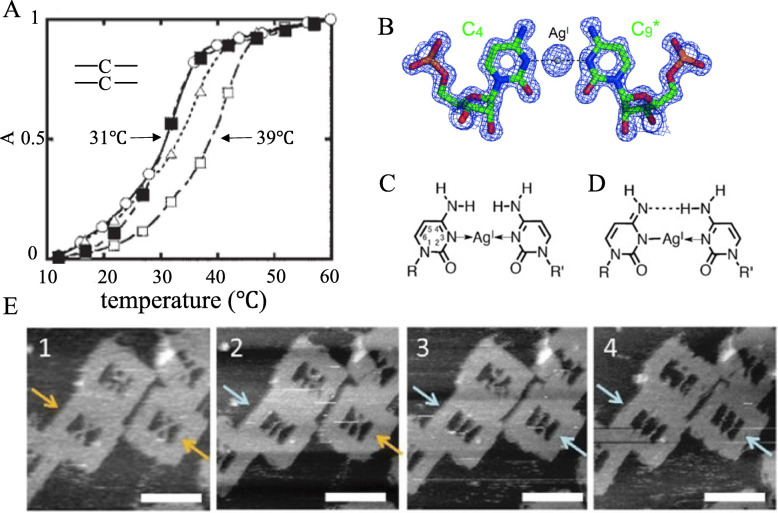
**A** Thermally induced transfer curve of DNA double-strands with C-C mismatch(reproduced with permission from Ref. [21],© Chem Commun 2008). **B** In the presence of Ag^+^, RNA forms a local structure of C-Ag^+^-C(reproduced with permission from Ref. [22],© Angewandte Chemie 2015). **C** N3-Ag^+^-N3 structure observed in Ag^+^-RNA complex(reproduced with permission from Ref. [23],© Chemistry 2016). **D** possible isomerized structure(reproduced with permission from Ref. [23],© Chemistry 2016). **E** AFM observation of the structural changes of double-stranded DNA on the DNA origami frame in the Ag^+^ solution. Yellow Arrows indicate connected strands(X-shape formation), Blue Arrows indicate separated shape.(reproduced with permission from Ref. [24],© Chemistry 2019)

In 2008, Ono [21] proved the existence of the C-Ag^+^ interaction by fluorescence resonance energy transfer, and proposed that silver ions can combine with the C-C mismatch in the DNA double strand to form a C-Ag^+^-C structure. The structure usually does not affect the conformation of DNA double strands, but it can increase the stability of the double strands. In the article, the authors found that in the presence of Ag^+^, the melting temperature T_m_ (melting temperature) of DNA double strands was 39 °C through the thermally induced transition profiles of DNA double strands containing mismatched C-C. In contrast, the melting temperature was down to 31 °C (as shown in Fig. 1A). Results reveal that Ag^+^ can effectively stabilize DNA double strands and has high specificity. And through the 1D 1H NMR, it was observed that after Ag^+^ was added, a new peak was generated in the imino proton region, and it was enhanced with the increase of the silver concentration. At the maximum peak strength, the molar ratio of Ag^+^/double-strand was about 1:1. This phenomenon explains the binding ratio of Ag^+^ and C-C mispairing.

However, the binding mechanism of C-Ag^+^-C is still unclear. In 2015, Kondo [22] analyzed the RNA lattice by X-ray(as shown in Fig. 1B) and proved that the mispairing of Ag^+^ and C-C was combined through the linear coordination of N3-Ag^+^-N3, and the conformation of the double-strand will not be affected.

Despite of the results, X-ray lattice analysis still have some problems [23]. For example, (1) the structure in Fig. 1C may be isomerized into the structure in Fig. 1D, in which the repelling amino-amino group is deprotonated by an amino group and then combined by hydrogen bonding. However, the structural discrimination between (C) and (D) in Fig. 1 is impossible in the crystal structure of the RNA duplex, because of the poor resolution of hydrogen atoms. (2) The typical helical arrangement of DNA and RNA may vary. Therefore, the chemical structure of C-Ag^+^-C in each duplex should be studied independently. (3) It should also be mentioned that the geometry of Fig. 1C may have steric/electrostatic repulsion between the amino groups of the paired cytosine residues. Therefore, if C-Ag^+^-C follows the c-type structure as Fig. 1, the way to avoid the amino-amino steric/electrostatic repulsion in the DNA duplex should be explained.

In 2016, Dairaku [23] proved that the connection site is N3 via 15 N NMR spectra, i. e., a C-Ag^+^-C structure is formed in the form of N3-Ag-N3. And the observation of the -NH_2_ signal on the N4 atom indicates that isomerization has not occurred.

In 2019, Xing [24] used DNA origami technique to design a DNA framework that can observe DNA morphological changes from single-molecule level. This method provides a multifunctional scaffold to place the substrate DNA strands which allows the system to be monitored at the molecular level. And then the single-molecule structure was observed using a high-speed atomic force microscope (AFM). The structural change is showed in the Fig. 1E. The dsDNA containing C-Ag^+^-C base pairs is found to dissociate from X-shape to separated shape, which suggests that the C-Ag^+^-C base pairs were not so stable in the DNA frame. The formation of the dsDNA in the DNA frame requires winding for helix formation from two topologically restricted DNA strands. Although the dsDNA containing C-Ag^+^-C pairs formed in solution, the structural stress attached in the DNA frame mechanically unwound the dsDNA.

Although remarkable progress has been made in recent year, there still remains the challenge of understanding the detailed conformation of C-Ag^+^-C. It is of crucial importance to use the liquid cell transmission electron microscope to dynamically observe the interaction between C and Ag^+^ in order to understand its insertion method.

## Application of silver-mediated base pairs

### Metal ion detection

**Fig. 2:**
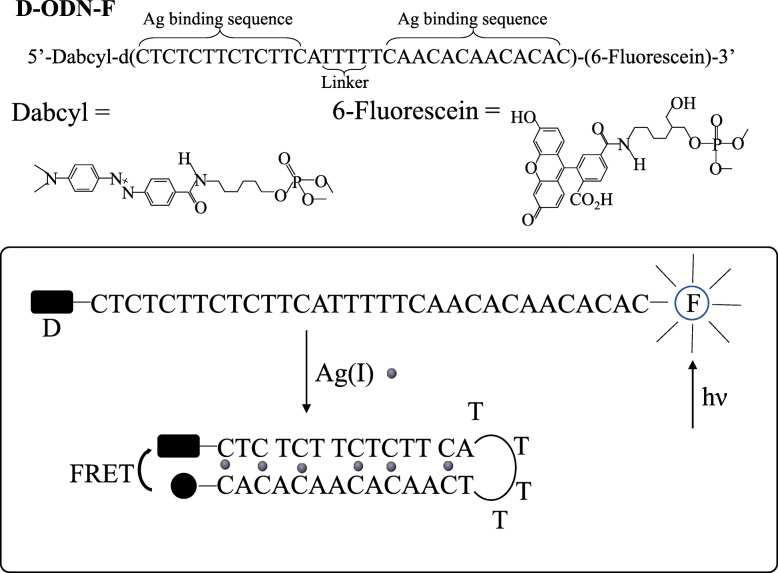
Schematic diagram of Ag^+^ sensor structure based on ODN and Ag^+^ induced C-Ag^+^-C formation to produce hairpin structure(reproduced with permission from Ref. [21],© Chem Commun 2008)

As early as 2008, Ono [21] et al. designed a DNA-based Ag^+^ sensor by employing the C-Ag^+^-C structure. An oligo-deoxyribon- ucleotide (ODN) has a fluorescent moiety (fluorescein, F) and a quencher (dabcyl, D) at the 3’end and 5’end, respectively. The structure and detection principle of this chain are shown in the Fig. 2. In the absence of Ag^+^, the DNA strands exist in a random coiled form; while in the presence of Ag^+^, the combination of C-Ag^+^-C enables the DNA strands to form a hairpin structure. In the former state, F and D are separated, leading strong fluorescence; in the hairpin structure, the ends of the ODN mutually interact, thereby enhancing the fluorescence resonance energy transfer (FRET) between the F and D components, and quenching the fluorescence. The undetected response to other metal ions proved the specific combination of Ag^+^ and DNA.

In 2009, Li and others [25] designed a colorimetric sensor for Ag^+^ detection by using the plasmon resonance of AuNPs. TEM was used to prove the influence of the binding of Ag^+^ and DNA on the aggregation of AuNPs. However, the binding state of Ag^+^ and DNA has not been observed. In their experiments, AuNPs served as probes. Once AuNPs are induced to aggregate, their plasmon peak at the wavelength of 520 nm decreases (the plasmon peak at 650 nm increases). The color of the solution changes from red to purple or blue. The strategy obtained a simple and sensitive DNA-based Ag^+^ colorimetric sensor which can be either label-free or labeled DNA and AuNPs probes.

**Fig. 3:**
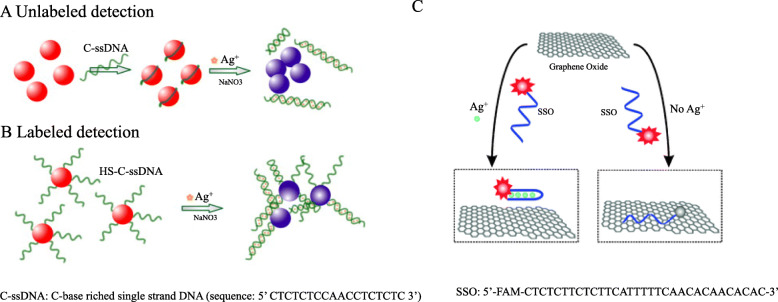
DNA-based Ag^+^ detection method for label-free (**A**) and labeled AuNPs colorimetric sensor (**B**) (reproduced with permission from Ref. [25],© Analytica Chimica Acta 2009); (**C**) Schematic diagram of Ag^+^ fluorescent sensor based on the targeted-induced conformational changes of silver-specific cytosine-rich oligonucleotides (SSO) and the interaction of fluorescent probes with graphene oxide. SSO: FAM-labeled silver-specific nucleotide probe(reproduced with permission from Ref. [26],© Journal of Luminescence 2010)

Label-free detection (Fig. 3A): C-rich single-stranded DNA entangles on AuNPs to form AuNPs/C-ssDNA complexes.

Labeled detection (Fig. 3B): Sulfhydryl-modified C-ssDNA (ie, HS-C-ssDNA) is covalently bound to AuNPs to form AuNPs-S-C-ssDNA complexes. In both methods, C-ssDNA or HS-C-ssDNA can enhance the stability of AuNPs to salt-induced aggregation. The addition of Ag^+^ reduces its stability due to the formation of C-Ag^+^-C complex. Therefore, the AuNPs colloidal solution containing Ag^+^ will aggregate into purple or blue, by which Ag^+^ can be detected. It was proposed by authors to use EDTA to eliminate the interference from other metal ions, achieving strong selectivity. This sensing platform not only expands the target library of DNA-based AuNPs colorimetric sensors, but also provides a simple and economical method for the Ag^+^ detection.

In 2010, Wen [26] and others developed a fluorescence sensor which quantifies the concentration of Ag^+^ (Fig. 3C). The experiment is based on the interaction between the conformational change of the DNA (SSO) fluorescent probe that specifically binds to Ag^+^ and the quenching effect of graphene oxide. SSO labeled with fluorescein (FAM) is used as a fluorescent probe for Ag^+^, and the SSO is rich in cytosine (C). In the absence of Ag^+^, SSO is in a flexible single-chain state. When Ag^+^ is added, Ag^+^ complexes with the cytosine bases of SSO form a rigid hairpin structure. Then, GO is added to selectively adsorb SSO that is not bound to Ag^+^ and quench its fluorescence, while SSO bound to Ag^+^ remains suspended and keeps fluorescence. Therefore, the Ag^+^ concentration can be quantitatively detected by the fluorescent intensity of SSO.

**Fig. 4:**
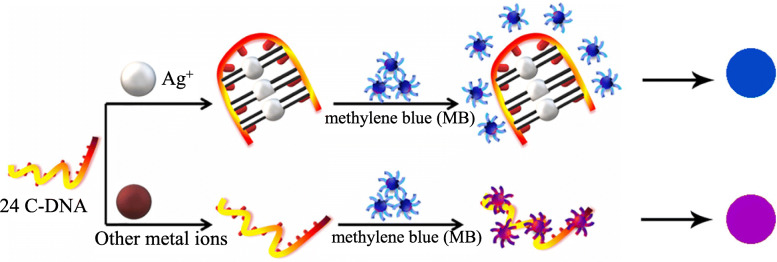
Principle of MB and 24C ssDNA colorimetric detection of Ag^+^(reproduced with permission from Ref. [9],© Microchemical Journal 2019)

In 2019, based on the specific interaction between methylene blue (MB) and cytosine C-rich single-stranded DNA (ssDNA, 24C), Xu [9] et al. proposed a method for simple and effective colorimetric method Ag^+^ detection(Fig. 4). When there is no Ag^+^, MB molecules will be absorbed to the surface of 24C, causing the color of MB to change from blue to purple; Contrarily, the formation of C- Ag^+^-C causes MB to be removed from the surface of 24C, and the color turns back to blue. Therefore, the selective detection of Ag^+^ can be achieved.

In 2012, Yan [27] et al. developed a highly sensitive Ag^+^ electrochemical sensing platform utilizing the specific interaction of Ag^+^ and C-C mismatches and the controllable assembly of MWCNTs (multi-walled carbon nanotubes). In the protocol, an unlabeled C-rich ssDNA was used as the probe. The presence of Ag^+^ induce the cytosine-rich DNA probe to fold from a random helical structure to a hairpin structure, and the hairpin structure cannot be wrapped on the surface of MWCNTs. Then the “bare” carbon nanotubes were assembled on the 16-mercaptohexadecano-ic acid self-assembled monolayer (MHA/SAM) modified gold electrode. Electron transfer occurred between the electrode and the electroactive indicator (FcCOOH). A peak current signal was then detected. Since the peak current on the gold electrode depends on the assembly of MWCNTs, which is related to the concentration of Ag^+^, Ag^+^ can be monitored indirectly. In this paper, the impact of Na^+^ concentration and incubation time in the mixed solution of MWCNTs/ Ag^+^/C-rich-ssDNA and MHA/SAM modified electrode on the measurement results was studied. Under the optimized setup, the addition of Ag^+^ shows a good electrochemical response and has good selectivity to other environmental metal ions. At the target Ag^+^ concentration as low as 1.3 nm, it is easy to measure the change in electron transfer efficiency using differential pulse voltammetry. The linear response range of Ag^+^ detection is 10-500 nM.

**Fig. 5:**
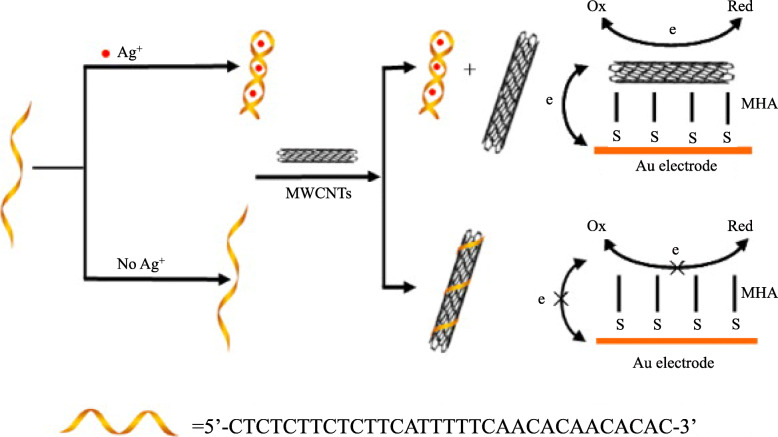
Schematic diagram of electrochemical analysis of Ag^+^ detection based on unlabeled C-rich ssDNA probes and MWCNTs controlled assembly(reproduced with permission from Ref. [27],© Talanta 2012)

The electrochemical detection of Ag^+^ based on MWCNTs and unlabeled Ag^+^-specific DNA is shown in the Fig. 5. First, the gold electrode was modified with dense MHA/SAM. The hydrophobic MHA/SAM separates the electrode from the aqueous solution. Therefore, the electron transfer between the redox solute and the electrode is blocked and electrochemical signal is not detected. When Ag^+^ is added, the cytosine bases of Ag^+^ and C rich-ssDNA probe form a rigid hairpin structure. The Ag^+^-C ssDNA complex cannot be adsorbed on MWCNTs effectually, and MWCNTs keep pristine and precipitate. The mixture is then immersed on the MHA/SAM electrode and assembled on the electrode through van der Waals forces and hydrophobic interactions to form “bare” MWCNTs. It will efficiently mediate the electron transfer between the electrode and the electroactive indicator. Then a strong redox current is generated. This is the open state. In the absence of Ag^+^, the C-rich ssDNA probe enables the interaction between DNA bases and MWCNTs through the π-π bond to form a MWCNTs/C-rich ssDNA complex that is stably dispersed in the solution. Due to the strong electrostatic and hydration repulsion between the ssDNA complex of MWCNTs/C and the negatively charged SAM, it will not be adsorbed on the MHA/SAM, maintaining the isolation of MHA/SAM. Background current signals is off.

**Fig. 6:**
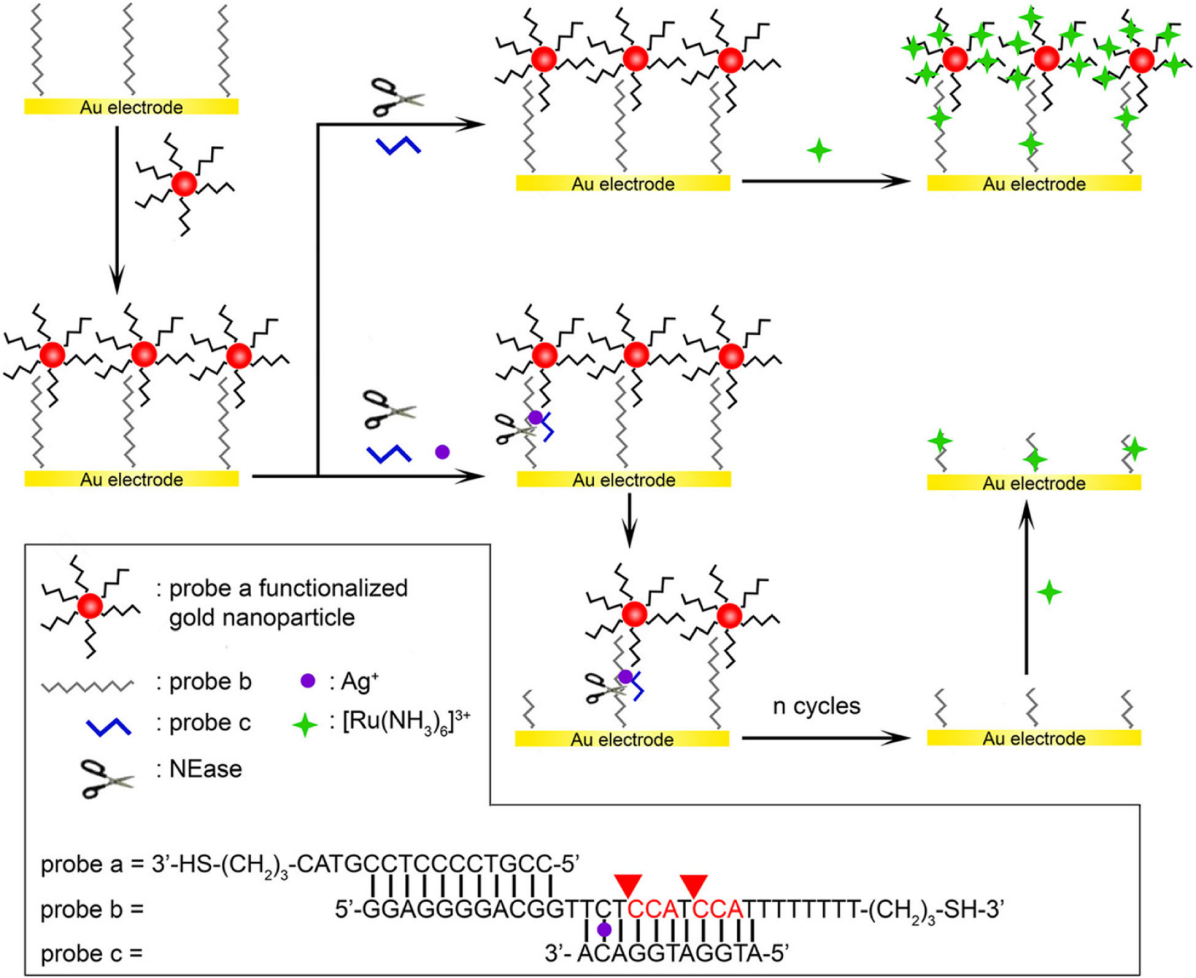
Diagram of dual signal amplification of gold nanoparticles and enzymatic cleavage(reproduced with permission from Ref. [28],© Analytical Chemistry 2013)

Miao [28] and others also designed an ultra-sensitive electrochemical sensor based on AuNPs and enzymatic cleavage of the dual signal amplification system to determine the concentration of Ag^+^. The basic principle is shown in the Fig. 6.

In the experiment, through Au-S bonds, gold nanoparticles was modified with the sulfhydryl-modified probe a while gold electrodes was modified with probe b. Then the two are mixed and put into the solution containing probe c and NEase enzyme. If there is no Ag^+^ in the solution, probe c cannot bind to probe b to form a NEase cleavage site, and the AuNPs are immobilized on the gold electrode; if Ag^+^ is added to the solution, probe c can bind to the probe a, NEase will cut off its binding site at this time to separate the AuNPs from the gold electrode. The experimental phenomenon is explained by electrochemical impedance spectroscopy, and eventually the detection limit of Ag^+^ is 400fM.

**Fig. 7:**
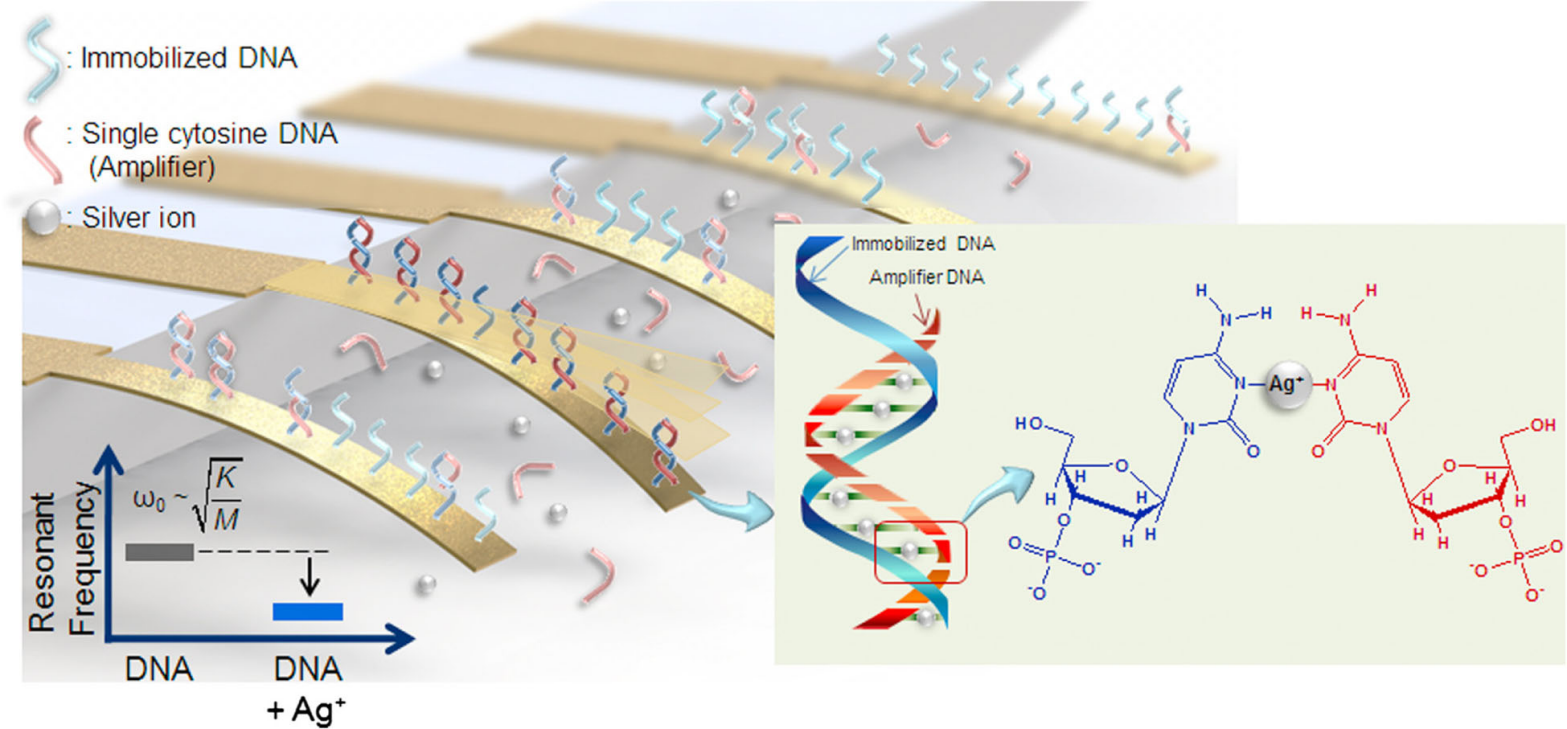
Schematic diagram of the silver ion-specific nucleotide coated oscillator (SSNO) (reproduced with permission from Ref. [29],© Biosensors and Bioelectronics 2013)

In 2013, Park [29] et al. designed an Ag^+^ detection method based on the principle of C-Ag^+^-C combination, silver-specific nucleic acid coated oscillator (SSNO) and sandwich structure array detect Ag^+^ (Fig. 7). The oscillator can detect Ag^+^ with a concentration of less than 1 nM.

**Fig. 8:**
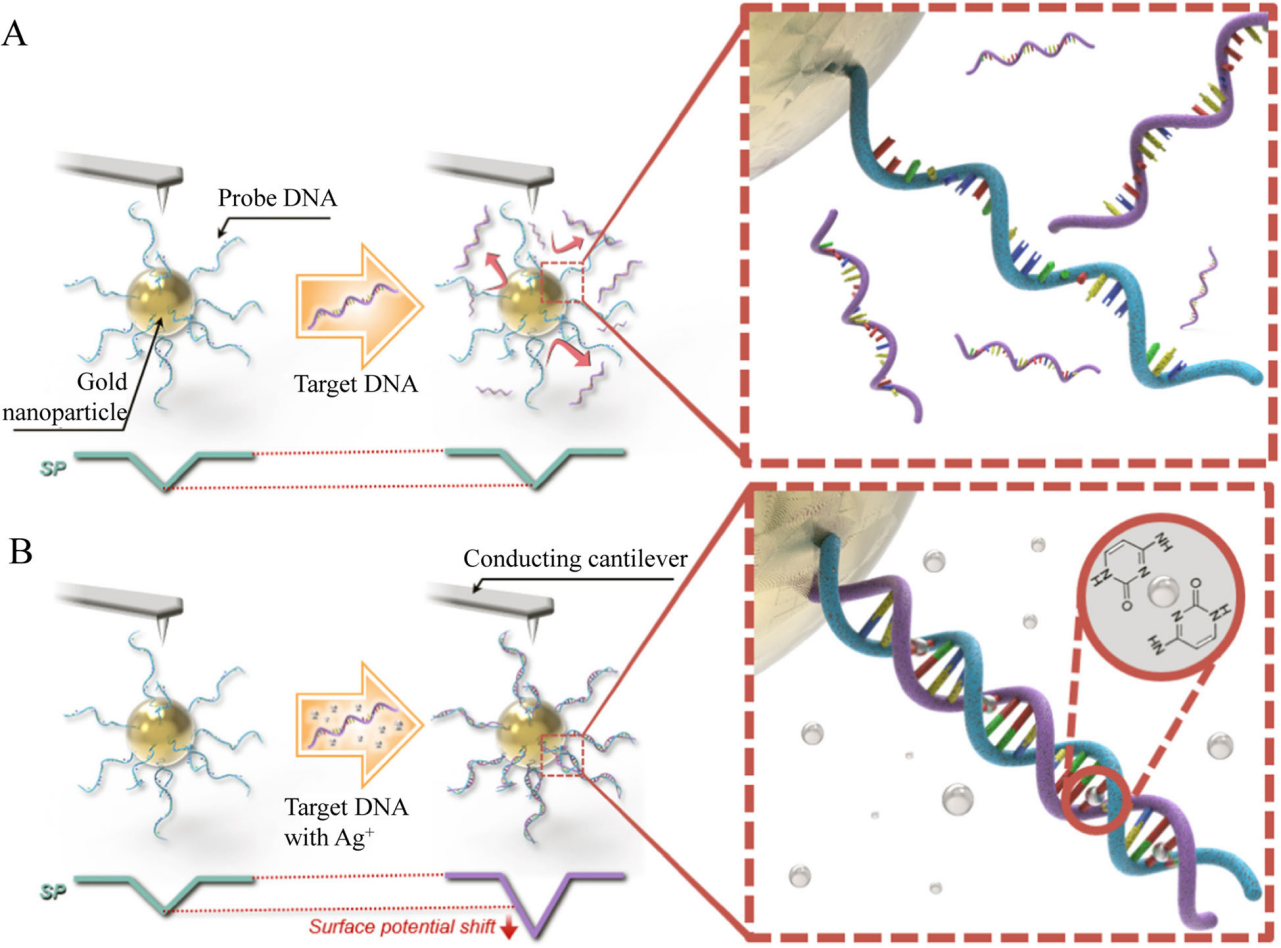
Schematic diagram of surface potential displacement of DCNP when KPFM is used to detect the presence of Ag^+^(reproduced with permission from Ref. [30],© Nanotechnology 2019)

Lee [30] used Kelvin probe microscopy (KPFM) to measure the surface potential of gold nanoparticles covered by DNA to detect Ag^+^. The basic principle is shown in the Fig. 8. First, in the absence of Ag^+^, no interaction happens between the probe DNA and the target DNA. Then the presence of Ag^+^ promotes the interaction between probe DNA (pDNA) and target DNA (tDNA). The hybridization of pDNA and tDNA forms double-stranded DNA on DCNP and induces surface potential shift of DCNP. Moreover, since there are ~ 100 pDNA on a DCNP and 4 C:C binding sites on each molecule, nearly 400 Ag^+^ can be combined, which greatly improves the sensitivity and dynamic range of detection. This platform is capable of extremely sensitive Ag^+^ detection (∼1fM) in a remarkably wide-range (1fM to1 μM).

**Fig. 9:**
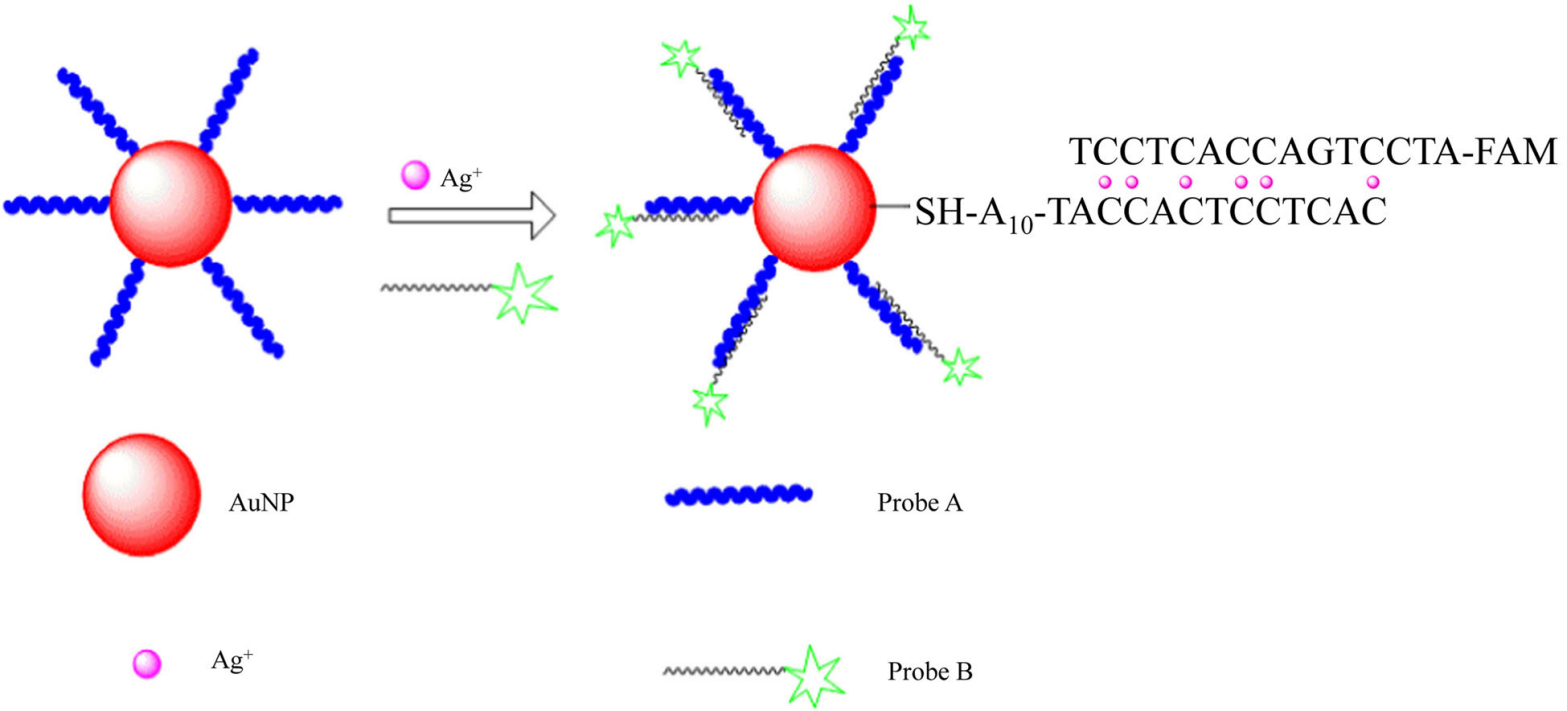
Schematic illustration of the assay for the detection of Ag^+^(reproduced with permission from Ref. [31],© Journal of Luminescence 2019)

Wang [31] et al. designed a fluorescence polarization sensor based on gold nanoparticles to monitor Ag^+^ effectively. The basic principle is shown in the Fig. 9. The fluorescence polarization (FP) is sensitive to the changes in the rotational movement of fluorescently labeled molecules. Small molecules rotate faster, having a smaller FP value; larger molecules rotate slower and have a larger FP. First, two complementary DNA strands probe A and probe B containing C:C mismatch are prepared. The -SH group of probe A can interact with gold nanoparticles and attach to the gold nanoparticles. Probe B is fluorescently labeled with FAM. Ag^+^ enables two probes (two CC mismatched DNA strands) to be stably paired during the formation of the C-Ag^+^-C complex, resulting in fluorescently labeled probe B and AuNPs surface stably attach. To further confirm the formation of C-Ag^+^-C complexes, differential scanning calorimetry (DSC) to conduct stability experiments on single-stranded DNA with/without Ag^+^ was applied. In the absence of Ag^+^, the melting temperature (Tm) of the mixed probe A and probe B solutions is ~ 53.6 °C, while addition of Ag^+^ elevates the Tm to ~ 63.5 °C. Therefore, the molecular volume increases significantly as well as the relaxation time and the FP value. Therefore, the quantitative detection of silver ions is achieved by using this strategy.

As shown in Table 1, we compared the lowest detectable concentration and working mechanism of the above sensors. The two commonly used methods are fluorescence spectroscopy and electrochemical detection. Their equipment requirements and operations are relatively simple, and the lowest detection concentration can be to the nanoscale. However, in order to achieve higher sensitivity, the detection method is more complicated, and the required instruments are also more expensive, which is inconvenient for rapid detection. The development of the subsequent silver ion detection sensor needs to increase the detection range while the method is simple, so as to realize simple operation and fast detection.

**Table 1:** Performance comparison of different sensors

Author	Working mechanism	lowest detectable concentration
**Ono** [21]	Fluorescence	About 10 nM
**Bingling Li** [25]	Colorimetric	0.59 nM
**Yanqin Wen** [26]	Fluorescence	5 nM
**Song Xu** [9]	Colorimetric	1 nM
**Genping Yan** [27]	Electrochemical	1.3 nM
**Peng Miao** [28]	Electrochemical	470 fM
**JinSung Park** [29]	Resonant frequency	1 nM
**Dongtak Lee** [30]	Kelvin probe force microscopy	1 fM
**Gongke Wang** [31]	Fluorescence	9.5 nM

### Logic gate and switch

**Fig. 10:**
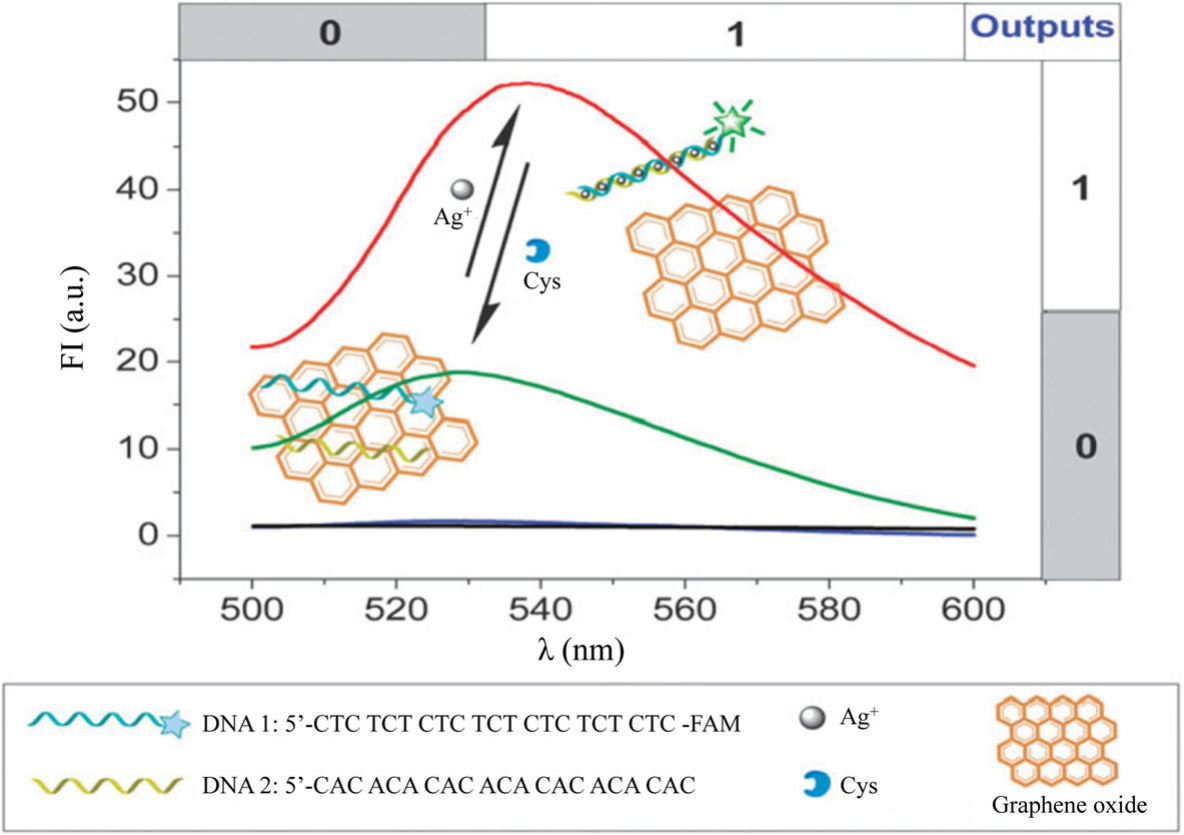
Ag^+^ and Cys detection principle based on graphene oxide and Ag^+^ characteristic nucleic acid(reproduced with permission from Ref. [32],© Chem Commun 2012)

Xie [32] et al. designed a Ag^+^ nano-switch using graphene oxide (GO) as a fluorescence quencher in 2012 (Fig. 10). The specific binding between two C-rich ssDNA and Ag^+^ is used. In the absence of Ag^+^, ssDNA is a flexible single strand. Through the accumulation of bases and GO, it is easy to form a stable GO/DNA complex in aqueous solution. Therefore, GO adsorbs ssDNA that is not bound to Ag^+^ and quenches the fluorescence of DNA labeled with FAM (fluorescein derivative). In the presence of Ag^+^, Ag^+^ may combine with the cytosine stable N3 nitrogen atom, resulting in the formation of C-Ag^+^-C complex. Not only the fluorescence intensity (FI) of FAM will increase, the fluorescence emission spectrum (λem) also undergoes a red shift.

Cysteine (Cys) is a sulfur-containing amino acid which plays an important role in the human body and is a potential neurotoxin. Cys is a strong Ag^+^ conjugator, which can be used to unfold the C-Ag^+^-C structure to convert double-stranded DNA into two ssDNAs. The ssDNAs will be adsorbed by GO, resulting in fluorescence quenching and blue-shift of fluorescence emission in which way Ag^+^ and Cys can be successfully detected. In addition, a dual-output fluorescent DNA suppression logic gate driven by GO-based Ag^+^/Cys is designed.

**Fig. 11:**
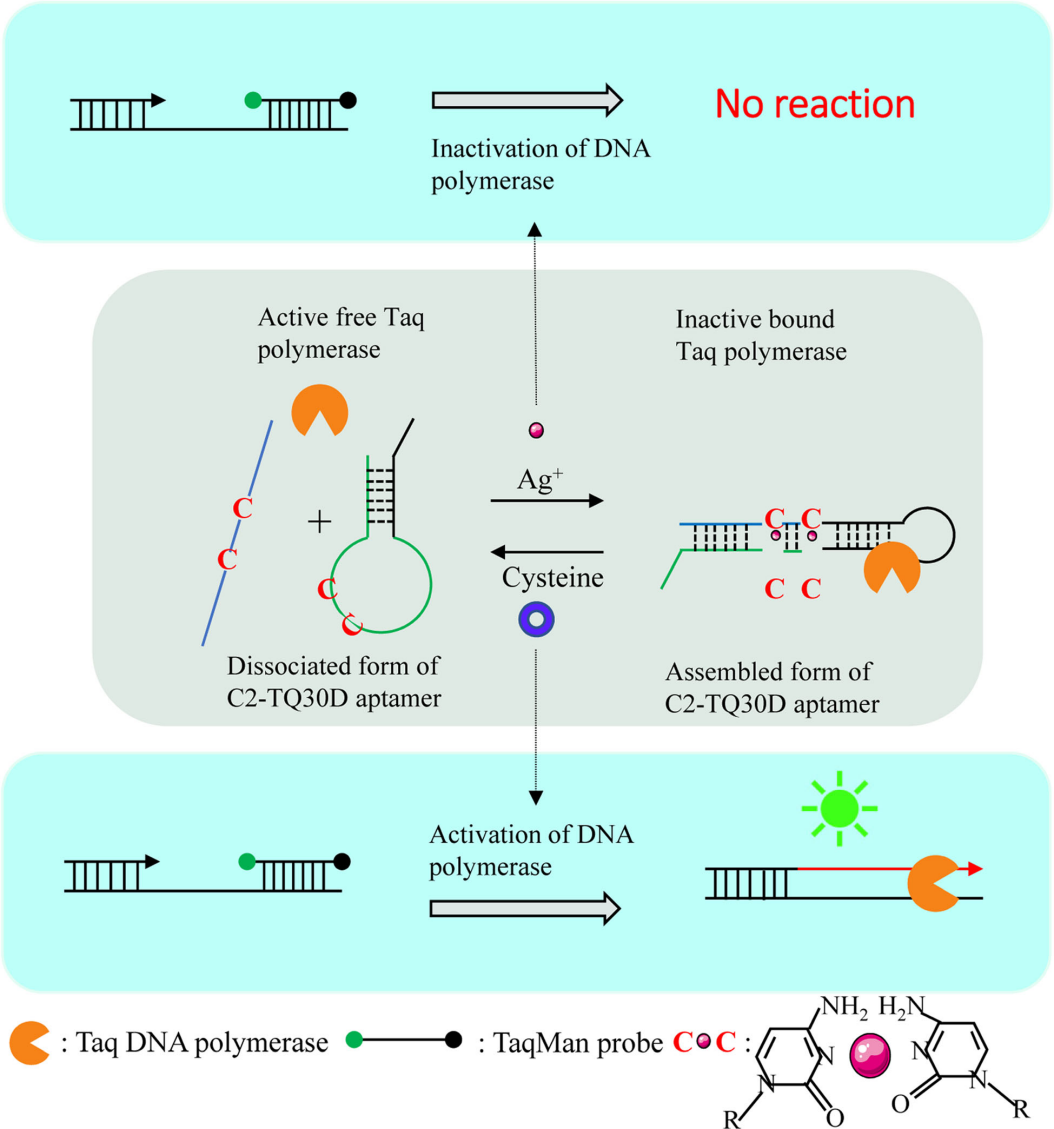
Schematic diagram of the reversible control of DNA polymerase activity by Ag^+^ and cysteine(reproduced with permission from Ref. [33],© Chemical Communications 2016)

Park [33] used the TQ30D DNA aptamer which can inhibit *Thermus aquaticus* DNA polymerase (*Thermus aquaticus* DNA polymerase) in the experiment (Fig. 11). It can effectively inhibit the activity of the DNA polymerase when the aptamer binds to the DNA polymerase. Ag^+^ can bind to the DNA aptamer, changing its structure, and therefore affecting its inhibitory effect on DNA polymerase. When cysteine is added, cysteine can bind to Ag^+^, thereby weakening the binding of Ag^+^ and DNA. The inhibitory effect on DNA polymerase ultimately realizes the reversible control of DNA polymerase.

**Fig. 12:**
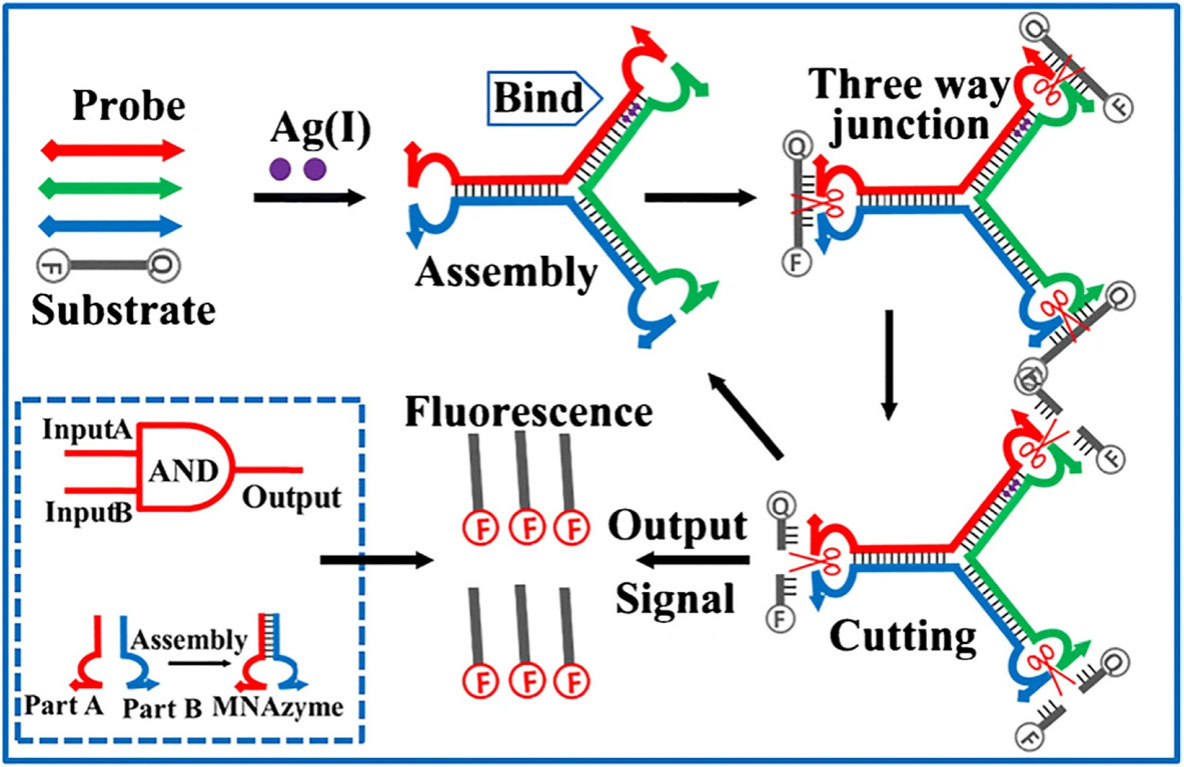
Schematic diagram of DNA-based Ag^+^ detection(reproduced with permission from Ref. [34],© Mikrochim Acta 2019)

In the same year, Xu [34] used the principle of combining catalytic core sequences (partzymes) to form a multi-component nuclease (MNAzyme), and designed a three-way junction nano-signal (3-WJ) to detect Ag^+^. The basic principle is shown in the Fig. 12. The signal reflecting concentration is amplified by the formation of the structure. The schematic diagram is shown in Fig. 11. The main components are three DNA probes A, B, and C (the MNAzyme is divided into two parts and placed at the 3’and 5’ends) and a substrate with a fluoresce and a quencher. In the absence of Ag^+^, the mismatch of C-C prevents the three probes from forming the 3-WJ structure as shown in the Fig. 12, and the fluorescence of the substrate cannot be released; while in the presence of Ag^+^, the formation of Ag^+^-C promotes the stable formation of 3-WJ structure. The ends of the three DNA probes are close enough to form three MNAzymes, which promote the decomposition of the substrate and emit fluorescence. The intensity of the fluorescence signal proved that Ag^+^ can promote the formation of a stable structure. The system was characterized by gel electrophoresis and AFM, which further proved the role of Ag^+^ and DNA.

**Fig. 13:**
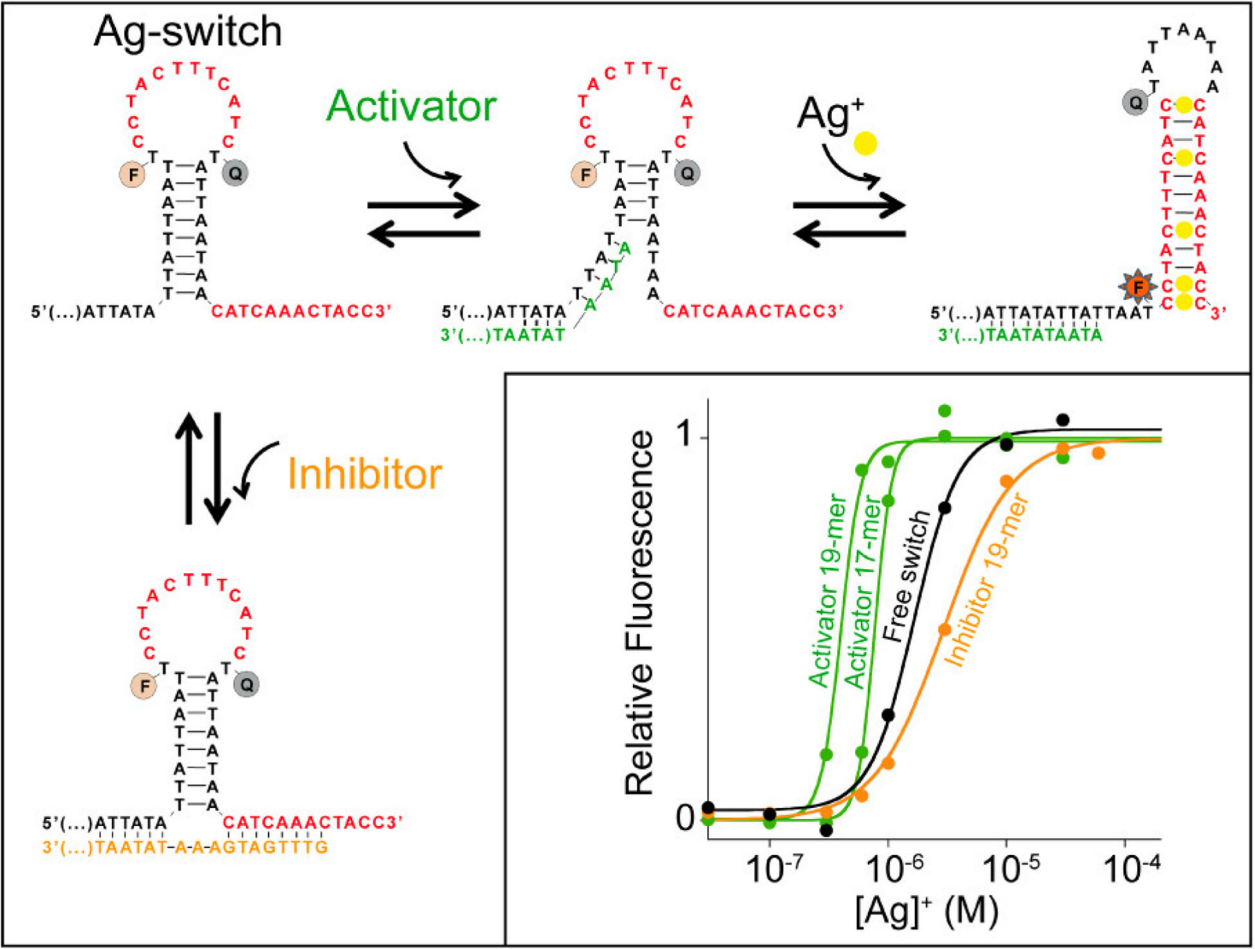
A tunable, silver(I)-activated switch employing silver(I)binding C−C mismatches as its recognition elements(reproduced with permission from Ref. [35],© Journal of the american chemical society 2013)

Porchetta [35] and others designed a switch by using the allosteric effect of DNA. This DNA sequence has two low-energy conformations, one of which cannot bind to any metal ions. In this conformation, the fluorescent agent and quencher on the DNA do not emit fluorescence; the other conformation contains metal ion specificity. The binding site can combine with metal ions to form a stable conformation and generate a fluorescent signal. The basic principle is shown in the Fig. 13. In order to prove the universality of this method, an adjustable switch is designed, which uses the C-C mismatch bound by Ag^+^ as the identification element. A single-stranded DNA sequence complementary to one or two allosteric tails in the unbound state is used as an allosteric effector to be activated (green curve) or be inhibited (orange curve) the switch and adjust its dynamic range. The black curve represents the combination of switches in the absence of allosteric effectors. It is noticed that through this switch, the presence of the activator resulted in a dose-response curve similar to that of collaboration. In the presence of inhibitors, this collaborative response is obscure. The switch concentration is maintained at 10 nM and the effector concentration is mantained at 20 nM.

**Fig. 14:**
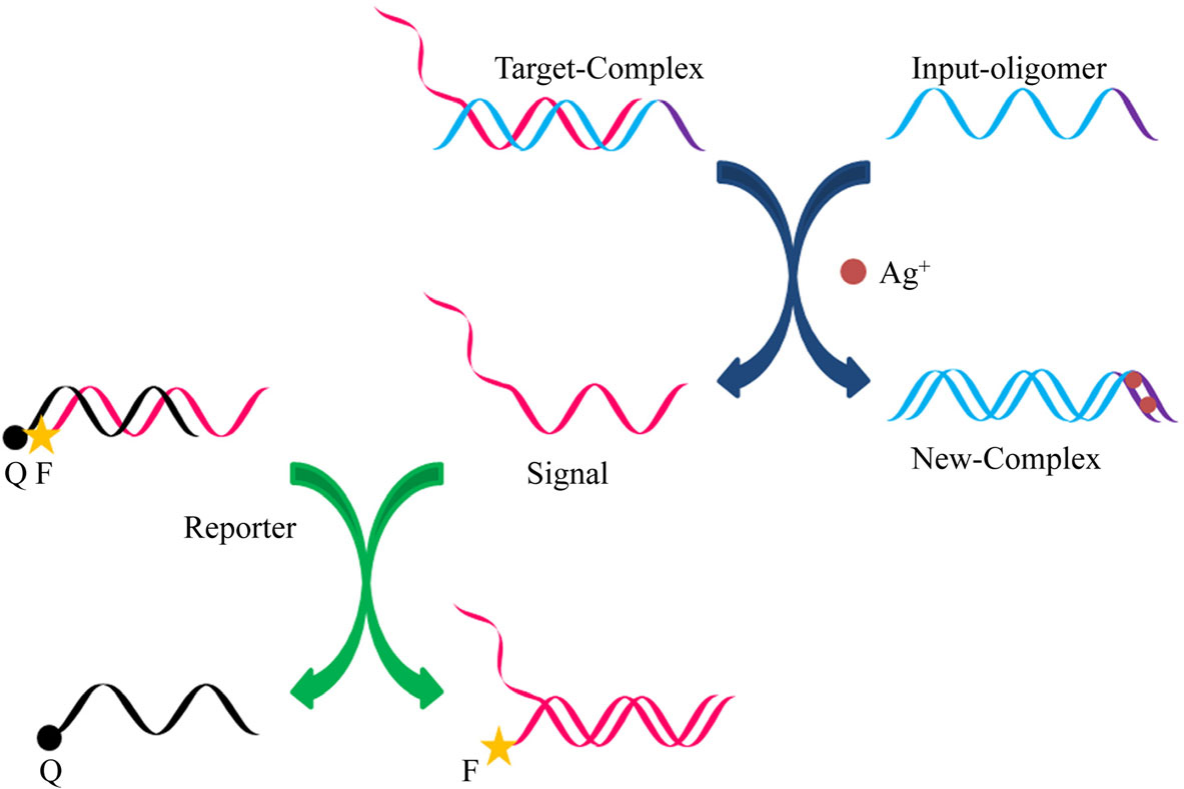
Principle of displacement reaction(reproduced with permission from Ref. [36],© PLOS ONE 2014)

Deng [36] et al. designed an experiment using Ag^+^ to control the DNA strand replacement reaction, which determined whether Ag^+^ can adjust the number of bases on the footing and the number of base mismatches on the footing to perform the DNA replacement reaction (Fig. 14). The “AND” and “OR” gates regulated by Ag^+^ and Hg^2+^ are designed. When Ag^+^ is introduced, the input DNA polymer interacts with the target complex to form a new complex and release the signal single strand; if an additional fluorescent reporter complex is used to perform fluorescence experiments, the released signal DNA single strand will interact with the reporter complex to separate the fluorescence and quencher to produce fluorescence when the input DNA polymer interacts with Ag^+^. Therefore, the progress of the strand displacement reaction can be observed by measuring the fluorescence intensity. The input and output are described as:

Input: Ag^+^/Hg^2+^. without Ag^+^/Hg^2+^ and with Ag^+^/Hg^2+^ are defined as “0” and “1”, respectively.

Output: fluorescence intensity, F0 and F respectively represents the fluorescence intensity of no metal ion and presence of metal ion. When (F-F0)/F0 is greater than 1, the output is 1; otherwise, the output is 0.

In a foothold containing 5 bases, there is a C:C and a T:T; in the absence of one or both of Ag^+^ and Hg^2+^, the DNA replacement reaction does not occur. When both Ag^+^ and Hg^2+^ are present, they form stable C-Ag^+^-C and T-Hg^2+^-T, respectively. The displacement reaction occurs, and the fluorescence intensity is significantly increased. Both the fluorescence intensity and the gel electrophoresis pattern show that the DNA replacement reaction will occur only when Ag^+^ and Hg^2+^ are present at the same time.

A foothold containing 6 bases includes a C:C and a T:T; in the absence of Ag^+^ and Hg^2+^, there are two mismatches, and the signal chain cannot be replaced; When there is one of Ag^+^ and Hg^2+^ or when both are present, at least one mismatch is stably connected, the ions promote the replacement of a single signal chain and enhance the fluorescence. Both the fluorescence intensity and the gel electrophoresis pattern show that the DNA replacement reaction will occur when either Ag^+^ or Hg^2+^ is present.

### DNA mutation detection

**Fig. 15:**
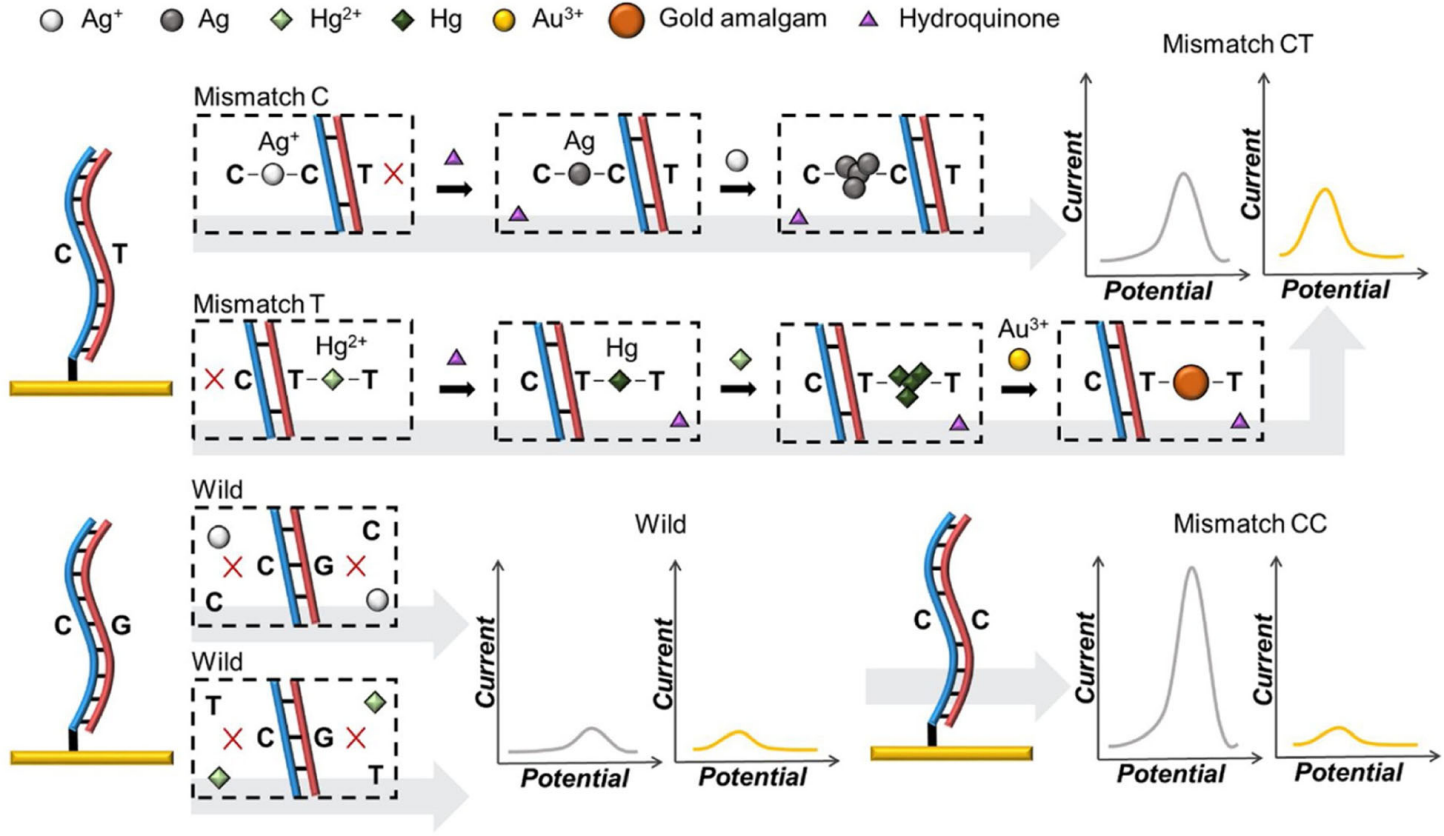
The principle diagram of DNA metal ion binding (C-Ag^+^-C, T-Hg^2+^-T) to detect SNP. Hydroquinone reduces metal ions. If it is mercury, gold ions are added to make a gold amalgam. DPV is used for detection, and mismatches are distinguished by comparing the peaks of silver and gold amalgam(reproduced with permission from Ref. [37],© Sensors and Actuators B: Chemical 2020)

The detection of single nucleotide polymorphisms (SNPs) has become an important technique for cancer identification. There is only one nucleotide difference between normal DNA and mutant DNA which makes it tricky to identify them. Therefore highly selective detection methods are necessary. Park [37] and others applied the principle that the mismatched combination of metal ions and DNA leads to changes in electrochemical properties, and used double pulse voltammetry (DPV) to establish a highly sensitive and selective SNP detection and identification method (Fig. 15). Utilizing the characteristics of cytosine-bound Ag ion and thymine-bound Hg ion, the experiment realized point mutation detection. After each metal ion is combined with the mutant DNA and the metal ion is reduced, the DPV signals is measured. Low-concentration of mutative DNA detection is achieved through external signal amplification.

### Nanowires and nanopatterns

**Fig. 16:**
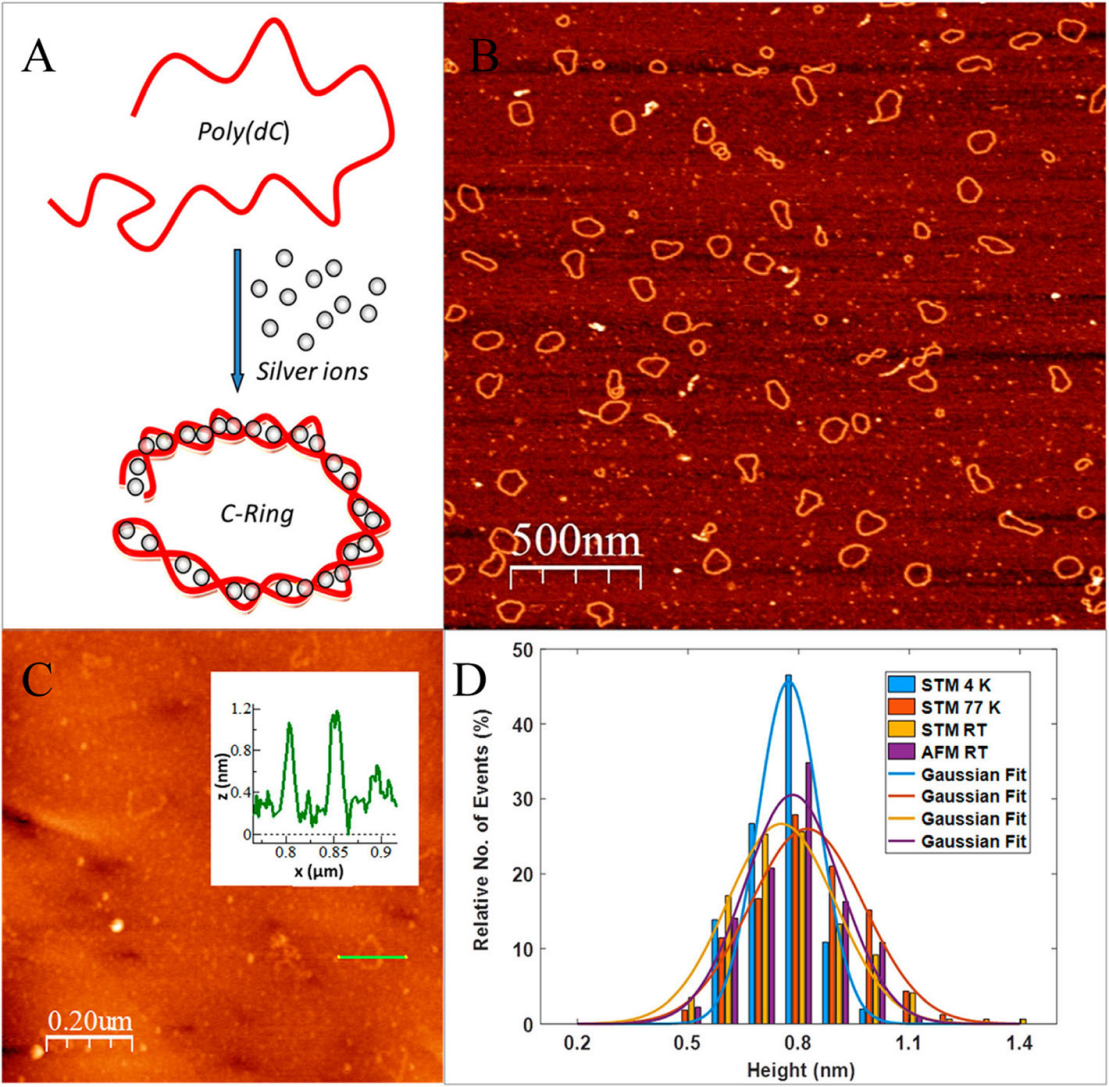
C-Ring morphology. **A** A long poly(C) single strand is folded into a C-Ring in the presence of Ag^+^ ions. **B** AFM image of C-Rings deposited on mica. **C** AFM image of C-Rings deposited on gold. Inset: a C-Ring cross-section, from which the local apparent height is obtained. **D** All four C-Ring height distributions, attained from AFM at RT, STM at RT, STM at 77 K, and STM at 4 K, yield the same average apparent height. (reproduced with permission from Ref. [38],© Nano Letters 2020)

Fardian-Melamed [38] used ~2000C bases and a 600 nm long single strand to interact with Ag^+^ in a recent study, and found that it would self-assemble into a 300 nm long C-ring (Fig. 16). AFM was used to characterize the morphology, and Scanning Tunneling Spectroscopy (STS) was used to reveal the electronic properties of the molecule. Ag^+^ inserted multiple cDNA strands have higher conductivity.

## Conclusions and outlook

Metal nanoparticles and ions, e. g., silver, have been widely used for its excellent antibacterial properties since ancient times. With the development of nanotechnology, silver has attracted wide attention not only as antibacterial components, but also in the field of nanodevices such as metal ion detection and logic gate switches. This article first introduces the discovery of the interaction between metal and DNA and its research methods, including nuclear magnetic resonance spectroscopy, X-ray diffraction, atomic force microscopy, etc., which proved that the binding specificity of silver and DNA mismatched C-C forms a more stable C-Ag^+^-C structure. Many researchers have developed various applications by applying this principle: colorimetry, fluorescence strategy, and electrochemical devices in the detection of metal ions. Reversible adjustable switches and logic gates have also been designed; The nanowires play an essential role in promoting the development of bioelectronic devices and nanomaterials.

Although the combination of C-Ag^+^-C has been studied in various ways, it is always proved by indirect means. With the development of liquid cell transmission electron microscopy and cryo-electron microscopy technology, perhaps it will provide more compelling and more intuitive evidence for this combination. A few trends can be discerned in current research on C-Ag^+^-C, all of which are associated with possible applications. An important direction is the optimization of nucleobases to enable the formation of duplexes comprising only metal-mediated base pairs, which not only significantly helps the construction of nanowires, but also enhances vias through the specific binding of metal ions to base pairs electrical signals are also very helpful to the detection of DNA sequences. It not only ameliorates the construction of nanowires, but also enhances vias through the specific binding of metal ions to base pairs electrical signals, which facilitates the detection of DNA sequences.

## Data Availability

All data generated or analysed during this study are included in this published article.
